# Artificial intelligence in retinal image analysis for hypertensive retinopathy diagnosis: a comprehensive review and perspective

**DOI:** 10.1186/s42492-025-00194-x

**Published:** 2025-05-01

**Authors:** Rajendra Kankrale, Manesh Kokare

**Affiliations:** 1 Department of Computer Science and Engineering, Shri Guru Gobind Singhji Institute of Engineering and Technology, Nanded, Maharashtra 431606 India; 2 Shri Guru Gobind Singhji Institute of Engineering and Technology, Nanded, Maharashtra 431606 India

**Keywords:** Hypertension, Hypertensive retinopathy, Artificial intelligence, Machine learning, Deep learning, Retinal image analysis

## Abstract

Hypertensive retinopathy (HR) occurs when the choroidal vessels, which form the photosensitive layer at the back of the eye, are injured owing to high blood pressure. Artificial intelligence (AI) in retinal image analysis (RIA) for HR diagnosis involves the use of advanced computational algorithms and machine learning (ML) strategies to recognize and evaluate signs of HR in retinal images automatically. This review aims to advance the field of HR diagnosis by investigating the latest ML and deep learning techniques, and highlighting their efficacy and capability for early diagnosis and intervention. By analyzing recent advancements and emerging trends, this study seeks to inspire further innovation in automated RIA. In this context, AI shows significant potential for enhancing the accuracy, effectiveness, and consistency of HR diagnoses. This will eventually lead to better clinical results by enabling earlier intervention and precise management of the condition. Overall, the integration of AI into RIA represents a considerable step forward in the early identification and treatment of HR, offering substantial benefits to both healthcare providers and patients.

## Introduction

Hypertension, which is also known as elevated blood pressure, is a chronic health condition that is characterized by steadily amplified arterial pressure [1]. This condition forces the heart to exert greater effort to circulate blood throughout the body, potentially leading to a range of serious health issues [2, 3]. Among these complications, heart disease, stroke, and kidney damage significantly affect quality of life. Hypertensive retinopathy (HR) is a notable complication of unmanaged hypertension [4, 5].

HR occurs when elevated arterial pressure causes structural changes in the retinal blood vessels, leading to various symptoms [6] including vision impairment, in which affected individuals experience blurred or reduced vision [7]. In addition, retinal hemorrhages or bleeding occur within the retina, along with swelling and the presence of cottonwool spots, which are fluffy white patches on the retina resulting from nerve fiber damage [8]. HR can progress to severe blindness or other serious ocular complications if not treated timeously.

Viewing HR requires greater detail to ensure that creases are minimized, allowing for a more accurate diagnosis based on fundoscopy [9]. The fundamental requirement is that the ophthalmologist must examine the back of the eye using a highly specialized device [10]. Examination by the ophthalmologist could expose arteriolar narrowing, hemorrhages, exudates, and optic nerve swelling [11–13]. The purpose of such investigation is to diagnose the degree and severity of hypertensive retinal damage for treatment to be properly targeted to the damage in each case [14, 15].

To control the damage already caused, one must know how to manage blood pressure effectively [16, 17]. In this respect, the best results can only be achieved by lifestyle improvements, e.g., balanced nutrition, regular exercise, and medication prescribed by an experienced physician. Immediate diagnosis and management are important for safeguarding vision and preventing the progression of HR and its complications [18]. Routine eye examinations are essential for patients with high blood pressure for the detection and timely management of this sign of retinal damage [19].

### Artery-vein classification

The use of these devices is automated for the artery-vein (A/V) classification in retinal analysis through artificial intelligence (AI) images prescribing arteries and veins. The system improves the quality of available and captured high-resolution retinal images obtained through preprocessing, that is, normalization, denoising, and contrast enhancement.

The implementation of vessel segmentation uses matched filters and deep learning (DL) representations, for example, the U-network (U-Net). The extracted features include the vessel width, branching patterns, intensity, and texture (e.g., local binary patterns), which can then be used for classification.

The identification of arteries and veins through automatic measurement and documentation is essential for correctly diagnosing high blood pressure [20]. An array of classifiers (e.g., support vector machine (SVM), which can also be voice-related to random forest) for classical machine learning (ML) and neural networks (NNs) (e.g., residual networks and dense networks) for categorizing vessels have isolated post-processing techniques for inaccuracy related to anatomical decrees and graph analysis.

For HR diagnosis, accurate A/V classification enables computation of the arterio-venous ratio (AVR) by measuring the average arterial and venous diameters, which are crucial for assessing vascular health. In addition, it facilitates the analysis of vascular abnormalities, e.g., increased vessel tortuosity, abnormal narrowing, and dilation, as well as the detection of lesions, e.g., hemorrhages and exudates. These AI-driven techniques enhance the diagnostic capabilities and provide critical quantitative metrics for the early detection and management of HR [21].

### AVR computation

The AVR computation in AI-based retinal image analysis (RIA) involves several technical steps that are designed to automate and enhance the accuracy (ACC) of this diagnostic metric [22]. The AVR is defined as the ratio of the average diameter of the retinal arteries to that of the retinal veins, and provides insights into vascular health and hypertension-related changes in the retina.

1 $$\mathrm{AVR}=\frac{\mathrm{Average}\;\mathrm{arterial}\;\mathrm{diameter}}{\mathrm{Average}\;\mathrm{venous}\;\mathrm{diameter}}$$

Equation 1 represents the formula for calculating the AVR. Compared with the traditional deterministic method (Eq. 1), the use of AI techniques such as explainable AI [23] and AI-based teleophthalmology applications for diagnosis enhances the AVR computation process by improving the ACC and robustness. While Eq. 1 is limited by its fixed approach, AI-based methods can adapt to variations in retinal images, providing more consistent and clinically useful results for HR diagnosis.

### Vessel tortuosity

Vessel tortuosity refers to the twisting and curving of blood vessels in the retina and is an important marker for assessing HR. This metric provides insights into structural changes in the retinal vasculature induced by hypertension [24]. It is crucial for assessing HR and providing a quantitative metric for vascular changes, as it aids in early detection and severity grading. The limitations of vessel tortuosity evaluation include variability in the algorithm performance across different image qualities and challenges in distinguishing pathological tortuosity from normal vascular variations without a clinical context.

### HR severity grading

The grading of HR severity in AI-based RIA involves categorizing the extent of vascular changes caused by hypertension. It uses quantitative measures, such as the AVR, vessel tortuosity, and presence of lesions, including hemorrhages and exudates. ML models categorize images into stages (e.g., mild, moderate, and severe) based on these features, aiding in the accurate diagnosis and monitoring of HR progression.

### Stages of HR

**Fig. 1:**
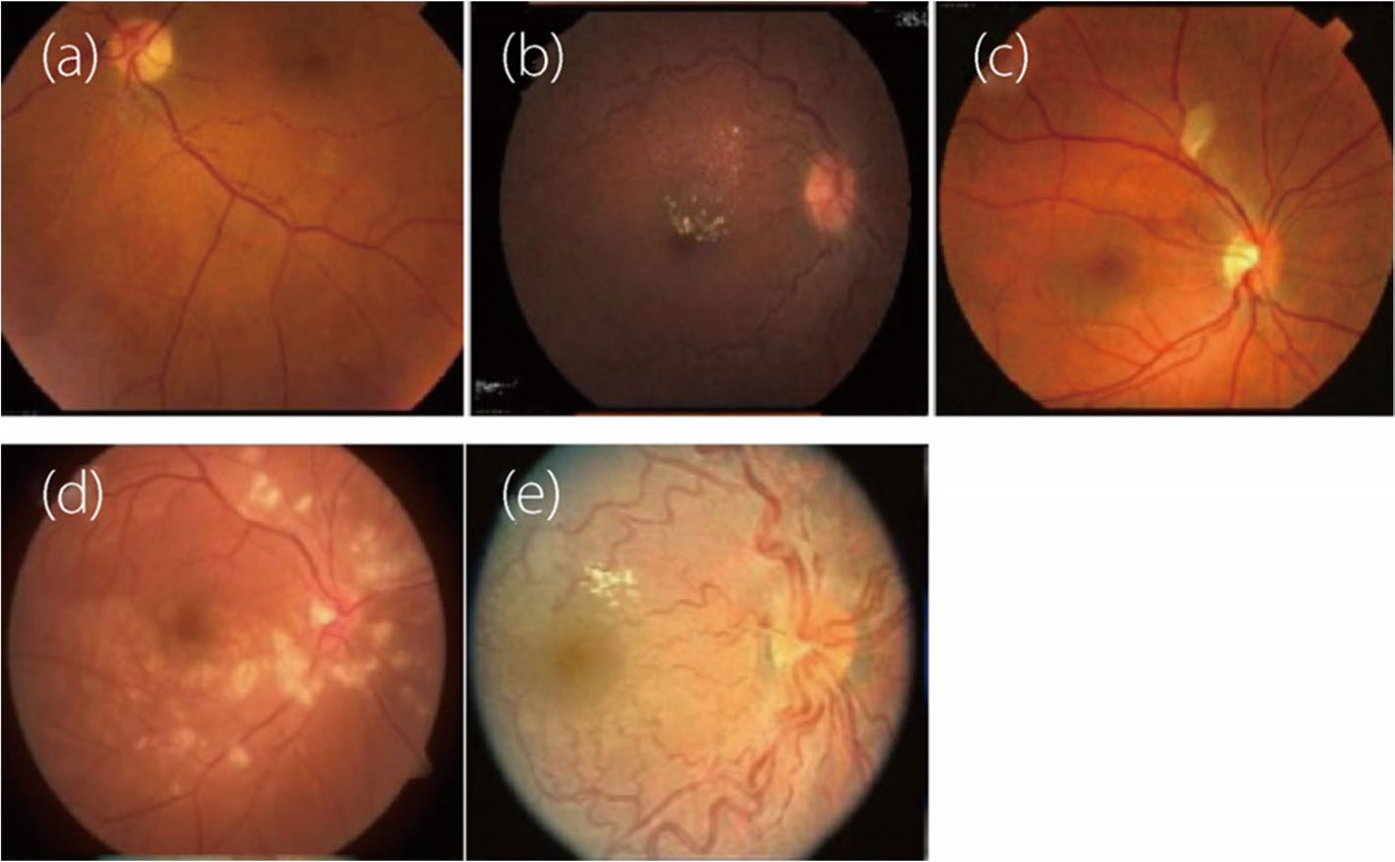
Visual representation of various phases of HR: **a **no sign of abnormality, **b **mild HR, **c** moderate HR, **d** severe HR, and (**e**) malignant HR

HR refers to retinal changes caused by chronic high blood pressure [25, 26], and progresses through five stages:

- No sign of abnormality: If the AVR is between 0.667 and 0.75, it is considered indicative of the first stage of HR, in which no abnormalities are typically observed. In this stage, retinal examination appears normal, with no visible signs of hypertension-induced damage. Blood pressure is elevated, but this has not yet affected the retinal vasculature.
- Mild HR: If the AVR decreases to 0.5, it is considered indicative of the second stage of HR, which is characterized by moderate narrowing of the retinal arterioles. Early signs include subtle changes in the retinal blood vessels, i.e., mild arteriolar narrowing or tortuosity.
- Moderate HR: If the AVR decreases to the 0.33 range, this indicates a moderate stage of HR characterized by increased arteriolar narrowing. More noticeable changes are observed in this stage, including arteriovenous nicking (in which the arterioles compress the venules at the crossing points) and increased arteriolar narrowing. Exudates (lipid residues), microaneurysms, and retinal hemorrhages appear.
- Severe HR: If the AVR value decreases to the 0.3–0.25 range, this indicates a severe stage of HR, in which significant damage to the retinal arterioles is observed. Significant damage occurs in this stage, including more extensive hemorrhages, cottonwool spots (areas of retinal ischemia), and extensive exudates. The risk of vision loss also increases.
- Malignant HR: If the AVR value decreases to 0.2 and below the 0.2 range, it indicates the malignant HR stage. This critical stage involves severe blood pressure elevation, which causes optic disc swelling (papilledema), widespread retinal hemorrhages, and exudates. It is a medical emergency requiring immediate intervention to prevent permanent vision loss. Figure 1 provides a visual representation of the various HR stages [27].

This review aims to advance the field of HR diagnosis by investigating the latest ML and DL approaches and highlighting their efficacy and potential for early detection and treatment. By analyzing recent advancements and emerging trends, this study seeks to inspire further research and innovation in automated RIA.

This study makes several key contributions to the field of HR diagnosis:

- This review examines ML and DL approaches to automated HR classification since 2019 by comparing datasets, preprocessing, feature extraction, segmentation, and advanced classification algorithms to highlight the latest and most efficient methods.
- This study examines automated tasks in retinal imaging, including A/V cataloging, AVR calculation, vessel tortuosity assessment, and HR sternness leveling, and reviews the literature on biomarkers as predictors of stroke risk. The research gaps and solutions for each area are discussed.
- Techniques are evaluated using diverse performance metrics to gauge their ACC and reliability. In addition, this study highlights recent breakthroughs and prospects, and offers insights into emerging trends for further research and development.

The remainder of this article is structured as follows: Research framework section outlines the research design, including the data acquisition and article search and filtering process. Latest advancements in medical imaging section discusses the latest advancements in classification and ACC. Latest developments in retinal vessel segmentation algorithms section describes recent developments in retinal vessel segmentation algorithms. Novel biomarkers identified from retinal imaging section focuses on the identification of novel biomarkers using retinal imaging. Performance analysis section presents the outcomes and discussion, and Conclusions section concludes the review and outlines potential future work.

## Research framework

This section describes the framework of the review, including the evaluation of a set of research papers, exploration of their origins, and detailed search criteria.

### Data acquisition

This review includes examples of researchers who have used AI to analyze retinal images for HR diagnosis.

**Fig. 2:**
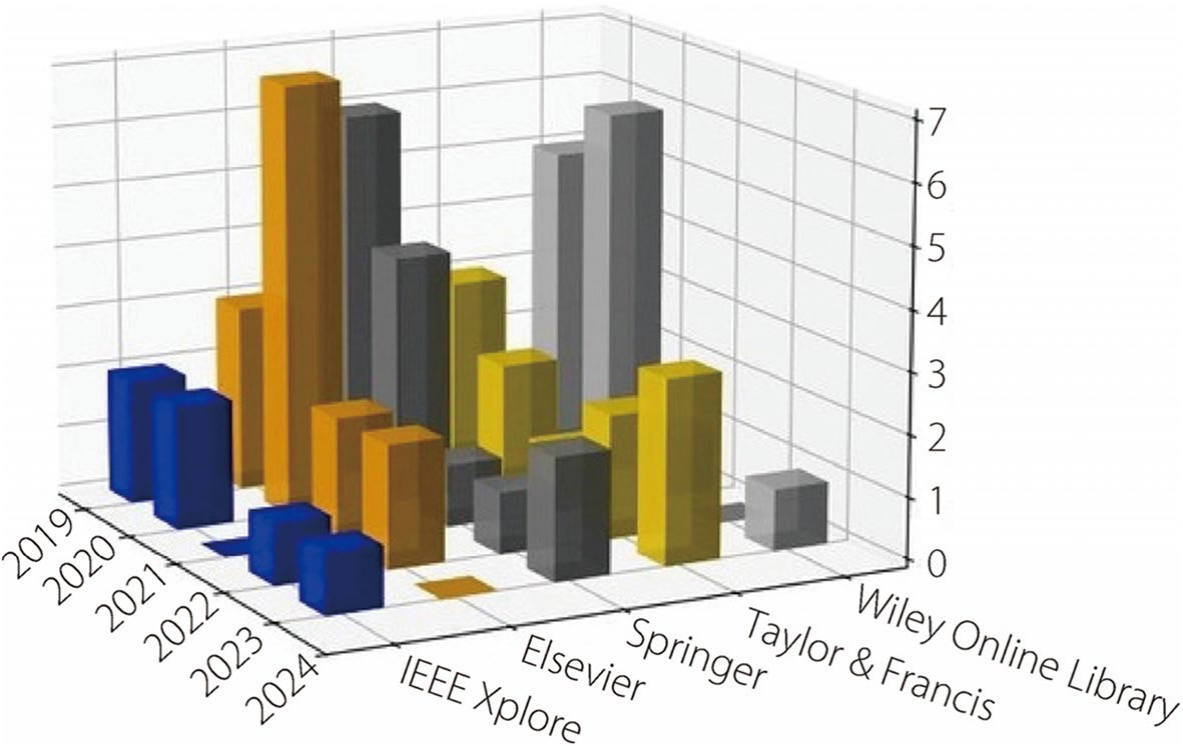
Sources of reviewed articles

As shown in Fig. 2, the most frequently used online library was Springer, followed by Elsevier, IEEE Xplore, Wiley Online Library, and Taylor & Francis.

**Fig. 3:**
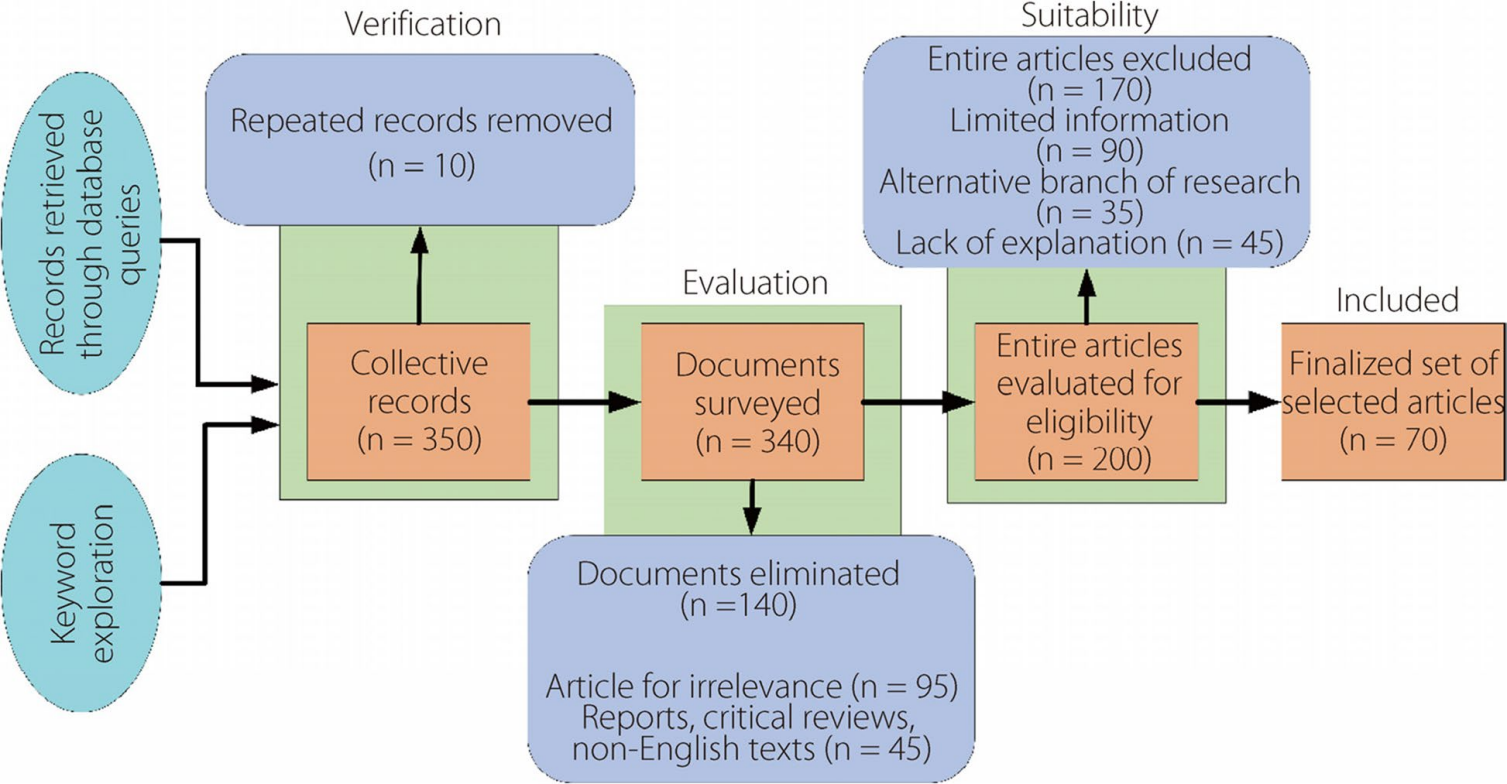
Process of article search and filtering

The article’s search and filtering process are shown in Fig. 3, started with retrieving 350 records through database queries and keyword exploration. After removing 10 repeated records, 340 unique records remained. These records underwent an evaluation phase in which 140 documents in the form of reports, critical reviews, or non-English texts were eliminated owing to irrelevance. This left 200 documents, which were then assessed for suitability, resulting in the exclusion of 170 articles for reasons, i.e., limited information, alternative research focus, or lack of explanation. Ultimately, this thorough filtering process yielded a finalized set of 70 articles for the study.

## Latest advancements in medical imaging

Innovations in this field have focused on refining image-acquisition technologies to capture higher-resolution and more detailed anatomical information. Moreover, there has been a concerted effort to integrate AI and ML algorithms into imaging analysis, enabling the automated detection, classification, and quantification of abnormalities with unprecedented ACC and speed.

What are the latest advancements in A/V cataloging, AVR computation, vessel tortuosity quantification, and HR severity grading systems that can enhance robustness and precision (PR)? Recent research in medical imaging has seen significant advancements aimed at enhancing the robustness and PR of various techniques. These methods include A/V cataloging, AVR calculation, vessel tortuosity quantification, and HR sternness leveling. The focus on improving the ACC and reliability of these methods suggests an ongoing effort to refine the diagnostic tools and methodologies to ensure that the models can provide more detailed and reliable insights into vascular health. Understanding the recent developments in these areas and their contributions to improved PR and robustness is crucial for advancing medical imaging practices. Recent AI advancements in RIA have improved A/V classification, AVR PR, vessel tortuosity quantification, and HR grading.

### Advancements in A/V classification

In recent years, great strides have been made in the AI applications of color fundus images for RIA to achieve more accurate classification in A/V duplication. Various DL methods have been applied, including convolutional neural networks (CNNs), which are fine-tuned to distinguish vessels based on their appearance with a higher percentage of PR. The training of these models involves numerous datasets annotated with arterioles or venules to learn differences based on morphology or appearance [28].

### Advancements in AVR computation PR

AI techniques have improved the ACC of AVR calculations by fully automating the measurement process with high ACC and reliability. DL models can robustly detect vessel edges, even in poor-quality images, owing to changes in illumination or noise in the image. This capability ensures consistent and precise AVR calculations, which are essential for assessing cardiovascular risk factors associated with HR [29].

### Advancements in vessel tortuosity quantification

AI algorithms enable precise quantification of vessel tortuosity from retinal images. However, traditional methods depend on manual or semi-automatic procedures that are prone to variability. Modern AI models, particularly DL architectures and specially designed algorithms, can automatically detect and measure vessel tortuosity with greater consistency across image qualities. Such a capability helps to detect and observe HR progression in a timely manner [30].

### Advancements in HR severity grading systems

AI has enhanced the grading of HR severity through the automated classification of retinal images according to disease severity. These systems utilize DL to analyze various features, including microaneurysms, hemorrhages, exudates, and arteriolar changes. The application of large-scale datasets and expert annotations allows AI models to attain high ACC in grading HR severity, enabling timely clinical intervention and monitoring [31].

### Survey of AI advancements in RIA improving A/V classification, AVR PR, vessel tortuosity quantification, and HR grading

Bhimavarapu [32] introduced an automated system known as fuzzy c-means clustering, which identifies HR in the initial phase of high blood pressure. Badawi et al. [33] suggested a technique for computing the AVR from the caliber extent of the A/V. Abbas and Ibrahim [34] proposed the novel densely connected Hypercolumn system to detect HR. Sajid et al. [35] proposed the Fully Automated System based on an optimized Inception model for HR classification. Soni et al. [36] proposed an Internet of things (IoT)-enabled federated learning-based HR classification model. Table 1 summarizes AI advancements in RIA.

**Table 1:** Comparative analysis of methods and models in retinal image processing and HR classification

Reference	Analysis type	Methodology	Dataset	Performance
Bhimavarapu [32]	Segmentation, AVR computation, classification	Fuzzy c-means clustering	DiaretDB0 dataset	ACC, F1-score, SN, SP
Badawi et al. [33]	AVR computation, vessel tortuosity, HR severity grading	Parr-Hubbard and Knudtson	RVM dataset	PR, kappa, SN, F1-score, and ACC
Abbas and Ibrahim [34]	Recognition and classification of HR	Deep residual learning, CNN	Imam HR dataset	SN, SP, AC, and AUC
Sajid et al. [35]	Recognition and classification of HR	InceptionV3 CNN and residual blocks	Pakistani hospital data	SN, SP, ACC, and AUC
Soni et al. [36]	A/V, HR classification	Radon vessel tracking algorithm, sequential network	AVN dataset	SN, SP, and ACC

*RVM* Retinal vessel morphometry, *SN* Sensitivity, *SP* Specificity, *AUC* Area under the curve

Overall, these advancements have enhanced the robustness against image quality variations and improved the PR of vessel tortuosity quantification and HR severity grading. These technologies hold promise for improving diagnostic reliability, facilitating early illness identification and advancing patient treatment in HR management.

**Fig. 4:**
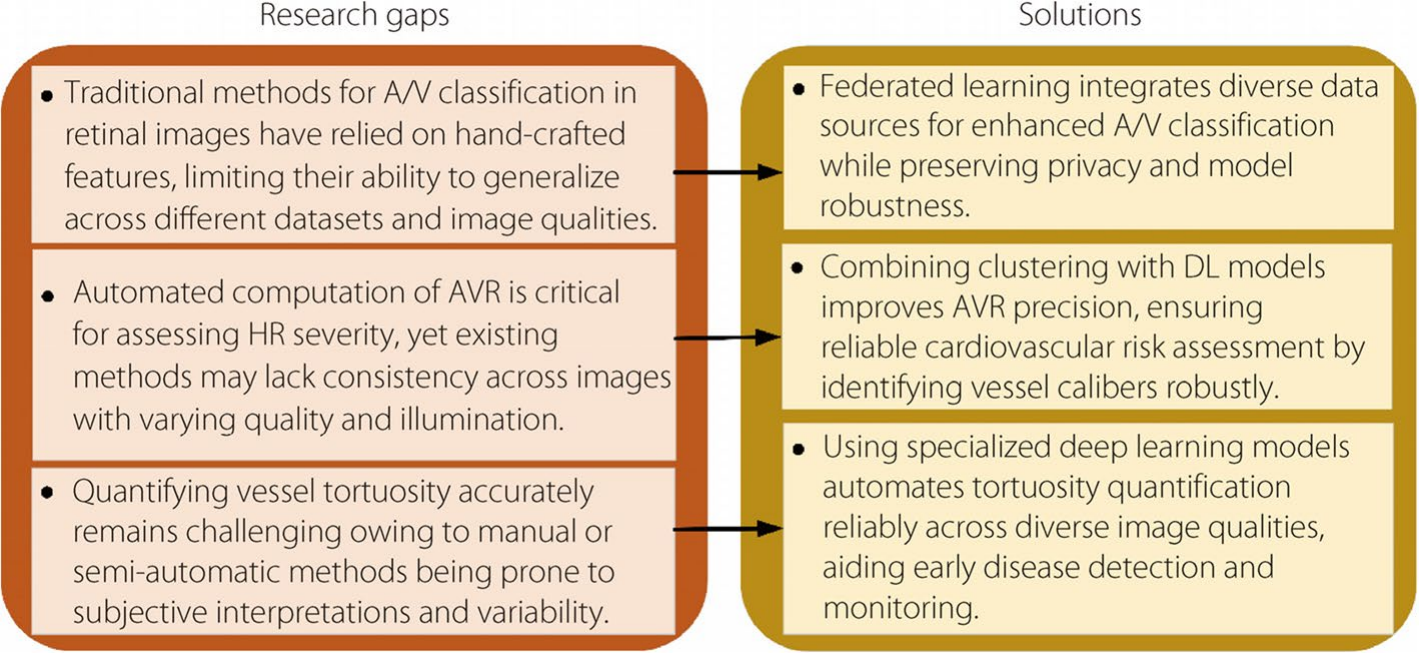
Research gaps and solutions for retinal image processing and HR classification

Figure 4 illustrates the research gaps and solutions for retinal image processing and HR classification. These innovations tackle issues such as variability in visual sharpness, enhanced ACC, and support for the prompt diagnosis and intervention of HR.

Typical A/V classification methods are limited by handmade features, making them less useful in different situations. To address this, automatic DL can improve the ACC and work better with different datasets and image qualities. In addition, federated learning can be applied to improve A/V sorting using different types of data while maintaining privacy. Grouping objects with DL models helps to make AVR measurements more precise, which is key to identifying heart health risks. Special DL models should be used to achieve accurate measurements, even when images appear different, which will help to avoid the problems of manual measurement. These new methods are very accurate, can be used in many locations, and are more applicable for real-world medical use.

## Latest developments in retinal vessel segmentation algorithms

New retinal vessel segmentation algorithms have markedly improved the ACC and reliability of HR detection. For example, hybrid models yield better segmentation outcomes by hybridizing the best aspects of classical methods with current DL techniques. One highlight of these models is leveraging the strengths of classical approaches and advanced capabilities of DL in a more precise and robust form of analysis [37].

How have the recent advances in retinal vessel segmentation algorithms improved the ACC and reliability of HR detection? Are there any hybrid models that combine traditional and DL methods for enhanced segmentation? Recent advancements, primarily driven by DL techniques, have significantly enhanced the ACC and reliability of HR detection. CNNs are particularly effective because of their capacity to capture hierarchical features, resulting in precise separation of retinal vessel networks. The U-Net model, which is used extensively for medical image segmentation, offers exceptional performance by efficiently learning high-level and detailed features through its encoder-decoder structure.

Generative adversarial networks further enhance the segmentation ACC by generating high-quality realistic images that improve the model training [38]. Attention mechanisms focus on important features in the image, improving the partitioning of delicate and intricate vessel networks, which is crucial for accurate HR diagnosis. Domain adaptation and transfer learning facilitate models in using pretrained weights that are derived from large-scale datasets, thereby enhancing the performance even with limited annotated retinal images.

Hybrid models that combine traditional and DL methods have also been developed to enhance segmentation. These models integrate conventional image processing techniques with advanced DL algorithms, leveraging the strengths of both approaches.

### Survey of advances in retinal vessel segmentation algorithms and hybrid approaches

Yan et al. [39] proposed a DL approach for retinal vessel caliber measurement, which surpassed existing semi-automatic software in terms of speed and convenience. Sanjeewani et al. [40] introduced a U-Net system to partition retinal blood vessels from fundus eye images. Joy et al. [41] proposed a new hybrid differential evolution approach for completing programmed retinal vein segmentation using NNs, SVM, and the micro firefly optimization algorithm (MFOA). Singh et al. [42] proposed a DL-based technique for blood vessel segmentation. They customized and fused two accessible networks: the residual network and attention network. Kumar and Gupta [43] proposed the new enhanced fuzzy c-means (EFCM) clustering scheme for vessel classification.

Table 2 presents a comparative analysis of the retinal vessel segmentation and hybrid techniques. Traditional methods, which frequently involve edge detection and morphology-based techniques, provide robust initial segmentation that can be refined using DL models. This combination leads to improved segmentation ACC and reliability, thereby facilitating superior HR detection and diagnosis.

**Table 2:** Comparative evaluation of retinal vessel segmentation and hybrid models

Reference	Analysis type	Methodology	Dataset	Performance
Yan et al. [39]	Vessel segmentation, arteriovenous classification	DL	Shougang study data, IDRiD	Error rate, SN
Sanjeewani et al. [40]	Preprocessing, segmentation	U-Net	DRIVE	SN, SP, ACC, F1-score
Joy et al. [41]	Vessel segmentation, classification	MFOA, NN, SVM	DRIVE, STARE, HRF	SN, SP, ACC
Singh et al. [42]	Vessel preprocessing, segmentation	Residual network, attention network	STARE	SN, SP, ACC, F1-score, PR, AUC
Kumar and Gupta [43]	Vessel preprocessing, segmentation, classification	EFCM, DenseNet, ShuffleNet	DRIVE, STARE, HRF	SN, SP, ACC, F1-score, PR, mean squared error, root mean squared error, and mean absolute error

*IDRiD* Indian diabetic retinopathy image dataset, *DRIVE* Digital retinal images for vessel extraction, *STARE* Structured analysis of the retina, *HRF* High-resolution fundus

**Fig. 5:**
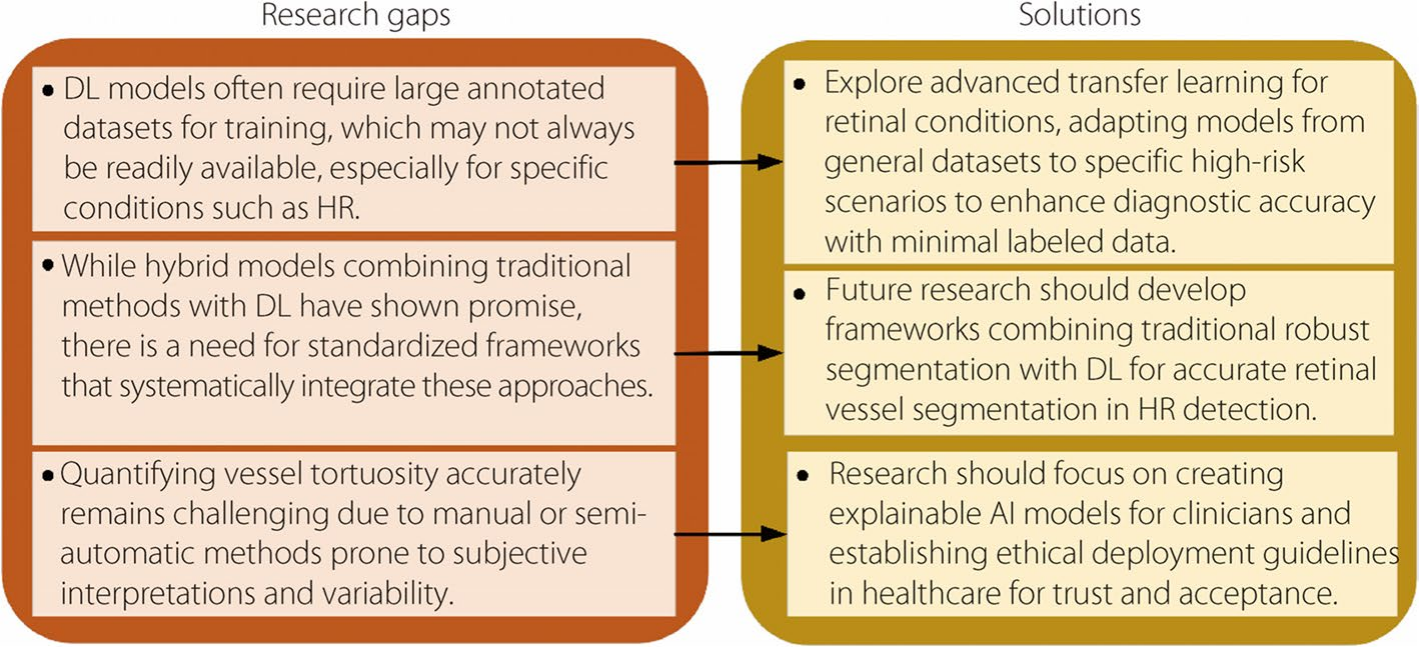
Literature gaps and proposed solutions for vessel segmentation methods

Figure 5 shows the research gaps and solutions for vessel segmentation methods. Advances in AI in RIA for HR with DL and hybrid models provide solutions, i.e., transfer learning, attention mechanisms, and clinical validation to enhance the diagnostic ACC and clinical utility and promote better ophthalmic healthcare outcomes.

Given the challenge of limited annotated datasets, especially for specific conditions such as HR, advanced transfer learning is suggested to adapt models from general datasets to high-risk scenarios, thereby enabling accurate diagnostics with minimal labeled data. In addition, hybrid systems combining traditional segmentation methods with DL should be developed to enhance vessel segmentation ACC in HR assessment. To address ethical considerations, the development of explainable AI models for transparency and clinical utility is emphasized, along with standardized guidelines for ethical AI deployment in healthcare to promote trust and acceptance. These solutions are designed to address the gaps in data availability, model interpretability, and ethical concerns that are critical for advancing AI integration into medical diagnostics.

## Novel biomarkers identified from retinal imaging

Novel biomarkers that are identified using retinal imaging offer insights into systemic health conditions. These biomarkers, including retinal vessel morphology, microvascular changes, and neuroretinal rim measurements, are non-invasive indicators of diseases, e.g., hypertension and diabetes. These features enhance early detection and monitoring, and potentially improve preventive healthcare strategies.

Which novel biomarkers identified from retinal imaging have shown promise in predicting stroke risk and how can AI leverage these in diagnostic models? Recent advancements in retinal imaging have revealed new biomarkers that can predict stroke risk. These markers, identified through a detailed analysis of eye images, provide new insights into the early signs of brain blood vessel dysfunction. AI has been integrated with these biomarkers to develop advanced diagnostic models. AI significantly improves the ACC of predicting stroke risk from eye scans, leading to improved prevention and personalized medical care. These biomarkers include microaneurysms, hemorrhages, vessel tortuosity, arteriovenous nicking, and retinal nerve fiber layer (RNFL) thickness.

### Retinal vessel caliber

Abnormalities in the retinal vessel caliber, i.e., arteriolar narrowing and venular widening, can indicate increased stroke risk. Narrowed arterioles suggest high blood pressure and vascular damage, whereas widened venules reflect systemic inflammation or poor blood flow, both of which are associated with a higher incidence of stroke [44].

### Retinal microaneurysms

Retinal microaneurysms cause damage to small blood vessels owing to weakening of the vessel walls, resulting in microvascular changes and diabetic retinopathy. They reflect a broader systemic vascular issue and increase the risk of stroke owing to impaired blood flow and changes in vascular integrity [45].

### Optic disc and cup morphology

Structural changes in the optic disc and cup, e.g., a change in the cup-to-disc ratio, indicate glaucomatous damage and other optic neuropathies. However, multiple studies have also connected this damage to cardiovascular diseases, e.g., stroke, highlighting a broader issue regarding damages to neurovascular health and systemic blood flow abnormalities [46].

### RNFL thickness

Thinning of the RNFL, which is the layer of nerve fibers in the retina, is usually associated with neurodegenerative disease. It indicates an underlying vascular problem and neurovascular health issues that increase the risk of stroke owing to compromised neural and vascular integrity [47].

### Retinal hemorrhages

Retinal hemorrhages are important because, as bleeding inside the retina, they may indicate prevailing significant systemic vascular pathology, e.g.,hypertension or diabetes. Hemorrhages usually indicate blood vessel instability and, consequently, an increased risk of stroke because they may even break a vessel, leading to impaired blood supply in brain tissues [48].

### Retinal pigment epithelium changes

Changes in the retinal pigment epithelium, i.e., the presence of drusen deposits, are typically observed during age-related macular degeneration. These changes could signal underlying vascular degeneration; inflammatory effects are a risk factor for stroke and are also indicative of compromised vascular health and function [49].

Geometric patterns in retinal vasculature: Deviations in the normal geometric patterns of the retinal vessels, i.e., altered fractal dimensions and increased tortuosity, are indicative of systemic vascular abnormalities. These changes predict stroke risk by reflecting broader issues with vascular health and blood flow efficiency throughout the body [50–52].

By incorporating AI, these biomarkers can be used to develop sophisticated diagnostic models, potentially enhancing the PR of stroke risk predictions based on eye scans. This advancement holds promise for improving preventive measures and tailoring medical interventions according to individual needs.

Leveraging AI in diagnostic models: AI can significantly enhance the predictive power and diagnostic capabilities when incorporating these retinal biomarkers [53, 54].

Image processing and feature extraction: AI algorithms, particularly DL models such as CNNs, automatically process retinal images to identify and quantify biomarkers with high ACC. These models analyze features, e.g., the vessel caliber, microaneurysms, hemorrhages, and optic disc morphology using high-resolution retinal scans [55, 56].

Predictive modeling: ML models integrate retinal biomarkers with other clinical data to create comprehensive risk prediction models. AI analyzes the patterns and correlations between retinal features and stroke incidence, improving the PR of risk assessment [57, 58].

Early detection and monitoring: AI-powered systems are used in regular screenings to detect subtle changes in retinal biomarkers over time, enabling early intervention and monitoring of at-risk individuals. The continuous learning capabilities of AI models allow them to improve over time, increasing their predictive ACC as more data become available [59, 60].

Integration with electronic health records (EHR): AI facilitates the integration of retinal imaging data with EHR, providing a complete perspective on patient health and improving the overall predictive power of diagnostic models. This integration supports personalized medicine approaches and tailors prevention strategies according to individual risk profiles [61].

Clinical decision support: AI-driven diagnostic tools assist clinicians by providing real-time analyses and risk assessments, thereby supporting more informed decision-making. Automated reporting systems highlight areas of concern in retinal imaging, streamline diagnostic processes, and reduce the burden on healthcare professionals [62].

By combining retinal imaging biomarkers with advanced AI techniques, it is possible to develop robust diagnostic models that enhance the early identification and avoidance of stroke, ultimately optimizing patient outcomes and reducing healthcare costs.

### Survey of novel biomarkers identified from retinal imaging

Soares et al. [45] introduced a multiscale algorithm designed for the automated identification of microaneurysms in retinal fundus images. Raveendran Susha et al. [48] proposed a method for predicting stroke using retinal vascular parameters. They conducted a regression analysis to identify relevant retinal features.

Hervella et al. [51] introduced the concurrent categorization of glaucoma and delineation of the optic disc and cup in retinal images. Arsalan et al. [53] introduced the pool less residual segmentation network (PLRS-Net), which was designed as a vessel segmentation model utilizing stride convolutions to enhance segmentation SN, effectively replacing traditional pooling mechanisms. Table 3 presents a comparative analysis of biomarkers identified from retinal imaging.

**Table 3:** Comparative analysis of biomarkers identified from retinal imaging

Reference	Biomarker type	Methodology	Dataset	Performance
Soares et al. [45]	Retinal microaneurysms	Multiscale algorithm	E-OphthaMA, Latim, Messidor, ROC training	SN, SP, F1-score, and AUC
Raveendran Susha et al. [48]	Retinal vascular	Logistic regression classifiers	Sree Gokulam Medical College and Research Foundation, Trivandrum	ACC, PR, SN, F1-score, and AUC
Hervella et al. [51]	Optic disc and cup	Multi adaptive optimization	REFUGE, DRISHTI-GS	AUC, Dice %, cup-to-disc ratio
Arsalan et al. [53]	Retinal vasculature	PLRS-Net	DRIVE, STARE, CHASE-DB1	SN, SP, AUC, and ACC

*CHASE-DB1* Child heart health study in England data base1

**Fig. 6:**
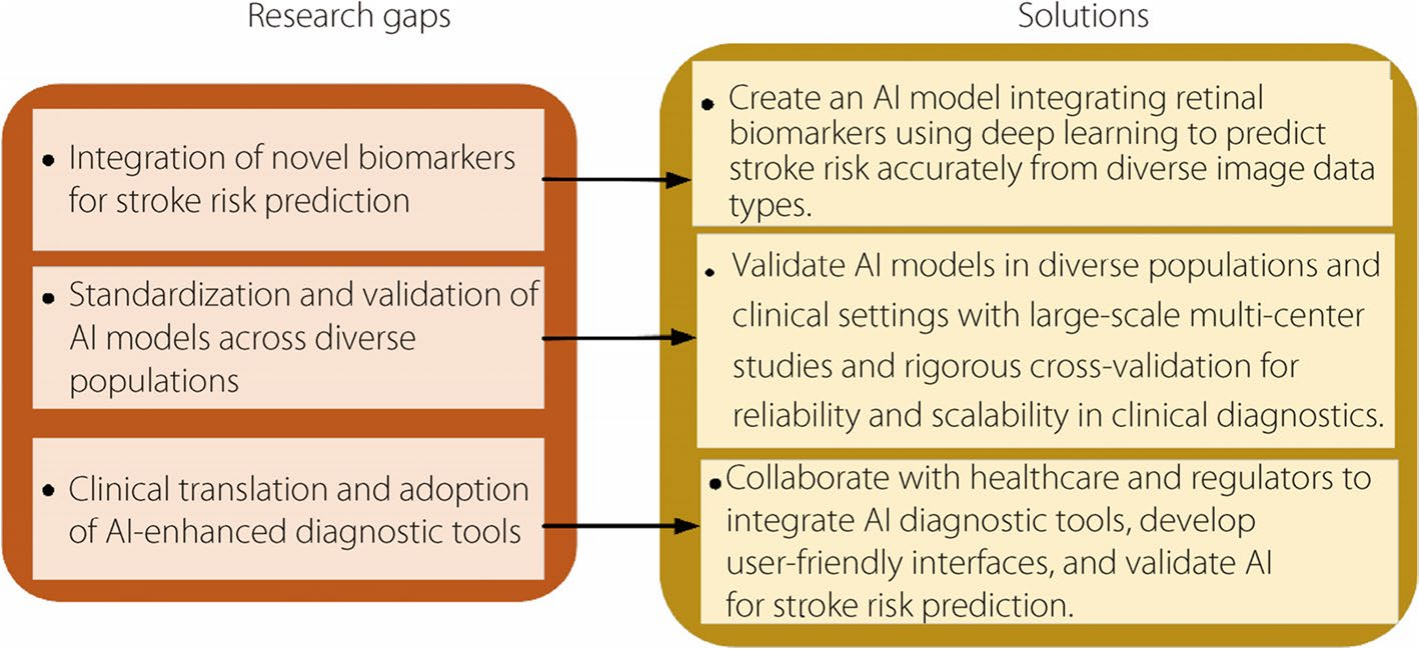
Research gaps and solutions for biomarkers identified from retinal imaging

Figure 6 shows the research gaps and solutions for biomarkers identified from retinal imaging. By addressing these research gaps through innovative AI methodologies and collaborative efforts, the field advances towards more effective early detection and management of stroke risk using retinal imaging biomarkers.

These solutions focus on developing a DL model that can integrate retinal biomarkers for accurate stroke risk assessment across various image data types. To ensure reliability and clinical relevance, large-scale multicenter studies with rigorous cross-validation are recommended for validation across diverse populations and clinical environments. In addition, collaboration with healthcare providers and regulatory bodies is emphasized to promote the clinical adoption of AI tools, with the aim of creating intuitive interfaces and ensuring regulatory compliance. These solutions target enhanced predictive ACC, validation across diverse cohorts, and smooth clinical integration, which are essential for practical, scalable, and ethically aligned AI diagnostics.

#### Overall research gaps

Critical knowledge gaps were elucidated when reviewing the state of the art in AI for HR diagnosis in RIA. These include requisites for more extensive sampling, variation, and annotation of datasets for training a strong AI model and challenges in validating AI effectiveness in real-world clinical settings. Algorithmic refinement should be aimed at improving the ACC across various patient demographics affected by disease severity, investigating improvements in AI in the integration of data from different imaging techniques for improving evaluations, and investigating the interpretability and transparency of AI in aiding clinical uptake. To advance predictive analytics and enhance treatment monitoring capabilities further, encourage interdisciplinary collaboration, and address regulatory and ethical considerations, there is a need to embark upon robust research programs in this field. Consequently, these loopholes must be filled to anchor AI in practice, so that high-risk HR diagnosis and management can be achieved, thereby improving patient outcomes and reducing the healthcare burden. The current section of this review presents a new strategy for addressing these challenges. This subsection introduces a new approach that targets the gaps in the proposed solutions using diverse and well-annotated datasets to train highly resilient AI models. This will lead to better patient outcomes and reduced healthcare costs.

#### Interpretability of models for HR diagnosis in RIA

For HR diagnosis, as in many other fields, it is essential that the AI models are interpretable for their clinical use. Whereas performance metrics, i.e., ACC, PR, SN, and SP provide a quantitative gauge of the reliability of the model in making predictions, interpretability presents the manner in which these models arrive at their diagnoses regarding the above metrics. Such an understanding, in turn, builds trust because it provides a certain level of information when assessing potential limitations of the model.

Recognition models, e.g., explainable AI, play a crucial role in determining which features (e.g., vessel caliber, tortuosity, hemorrhages, and other retinal abnormalities) are more influential in diagnosing HR. Explainable AI techniques, i.e., feature attribution and saliency maps, provide a visual explanation; thus, the specific areas or features of retinal images are explicitly clarified for model decisions. For example, saliency maps produce heat maps that are overlaid on retinal images, highlighting the regions on which the model focuses during the diagnostic process, i.e., narrowed arterioles or hemorrhage spots. This not only helps to confirm whether the model really works on clinically pertinent features but also permits clinicians to cross-reference these observations with observations of their own.

Embedding interpretability techniques into HR diagnostic models may further enhance the model transparency. For instance, feature attribution, i.e., SHapley Additive exPlanations or Local Interpretable Model-agnostic Explanations helps to quantify the individual contributions of features of the retinal image to the diagnostic result, thereby providing a cleaner picture of the “plan of thought” being used by the model. This categorically defines the possible biases or undesirable results so that they can be addressed most effectively in the quality control system.

## Performance analysis

This subsection examines diverse assessment criteria, i.e., the PR, SP, ACC, SN, and F1-score. These parameters collectively represent a thorough evaluation of the PR and reliability of the methods, facilitating an assessment of the efficiency of the proposed techniques.

Table 4 outlines the formulas employed for calculating various parameters. True positive (TP), false positive (FP), false negative (FN), and true negative (TN) denote the counts of the correctly identified unchanged pixels, unpredicted unchanged pixels, unpredicted changed pixels, and correctly identified changed pixels, respectively. Table 5 presents the execution analyses of various methods.

**Table 4:** Formulas for calculating performance metrics

Parameter used with formula				
Accuracy	Precision	Sensitivity	F1-score	Specificity
$ACC=\frac{TP+TN}{TP+TN+FN+FP}$	$\mathrm{PR}=\frac{TP}{TP+FP}$	$SN=\frac{TP}{TP+FN}$	$F1-score=\frac{2 precision*recall}{precision+recall}$	$SP=\frac{TP}{TN+FP}$

The reviewed literature on healthcare monitoring using IoT-quantifiable and qualitative presentation metrics is presented in Table 5. From the table, it is understood that a larger part of the research has focused on one fundamental metric, namely ACC, which assesses the proportion of accurately classified instances, whether positive or negative.

**Table 5:**
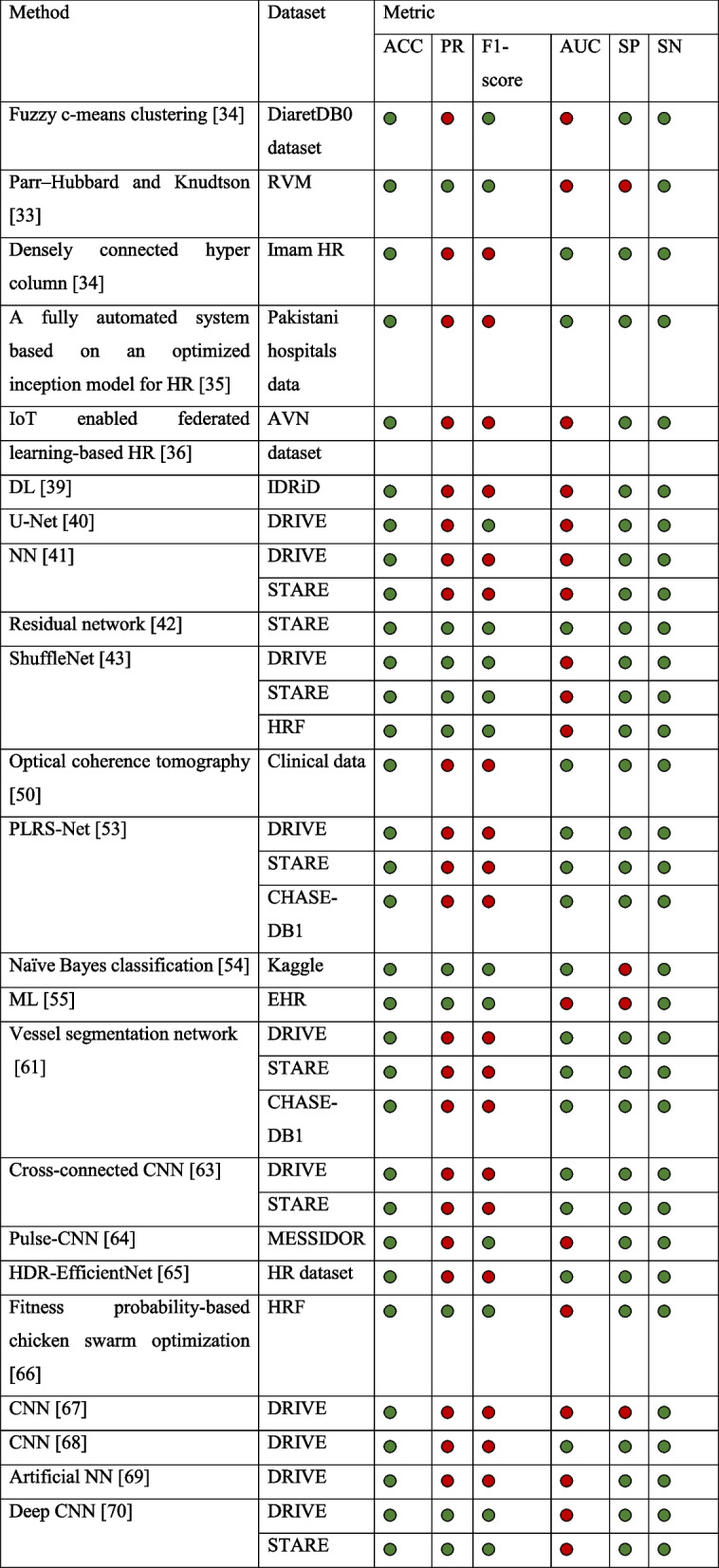
Assessment of different approaches [33–36, 39–43, 50, 53–55, 61, 63–70]

“  ” represents metrics that were reported in the reviewed article, “  ” represents metrics that were not reported in the reviewed article

Table 6 provides an overview of the datasets utilized in the reviewed studies, highlighting key details, i.e., the dataset size, training and test set distribution, and pixel size, as well as training parameters, i.e., the epochs, learning rate, and batch size.

**Table 6:** Summary of datasets used in AI-based HR diagnosis

Dataset	Total image	Pixel size	Training set	Test set	Epochs size	Learning rate	Batch size
DiaretDB0 [32]	2000	525 ✕ 525	1700	300	500	0.01	32
RVM [33]	504	2000 ✕ 2000	404	100	100	0.001	64
Imam HR [34]	4270	700 ✕ 600	2670	1600	100	0.01	32
Pakistani hospitals [35]	6000	700 ✕ 600	2280	3720	100	0.001	32
AVN [36]	1364	2592 ✕ 1728	1090	274	100	0.001	64
IDRiD [39]	516	4288 ✕ 2848	413	103	100	0.0001	32
DRIVE [40]	28	584 ✕ 565	24	4	50	0.01	64
STARE [41]	30	700 ✕ 605	24	6	50	0.01	64
HRF [43]	27	3888 ✕ 2592	23	4	50	0.01	64
CHASE-DB1 [53]	28	999 ✕ 960	14	14	10	0.00005	-
MESSIDOR [64]	1200	2304 ✕ 1536	960	240	-	-	-

The datasets used in the reviewed studies varied in terms of size, image resolution, and availability. These datasets typically consist of high-resolution retinal images that are suitable for HR diagnosis. The dataset sizes ranged from smaller sets, e.g., DRIVE (28 images), to larger sets, e.g., Imam HR and Pakistani hospital data, which contain over 4000 and 6000 images, respectively. Several datasets, including DiaretDB0, DRIVE, and MESSIDOR, are publicly available. These datasets were divided into training and test sets with different allocations, reflecting the diverse configurations used to evaluate the AI models for HR diagnosis.

**Fig. 7:**
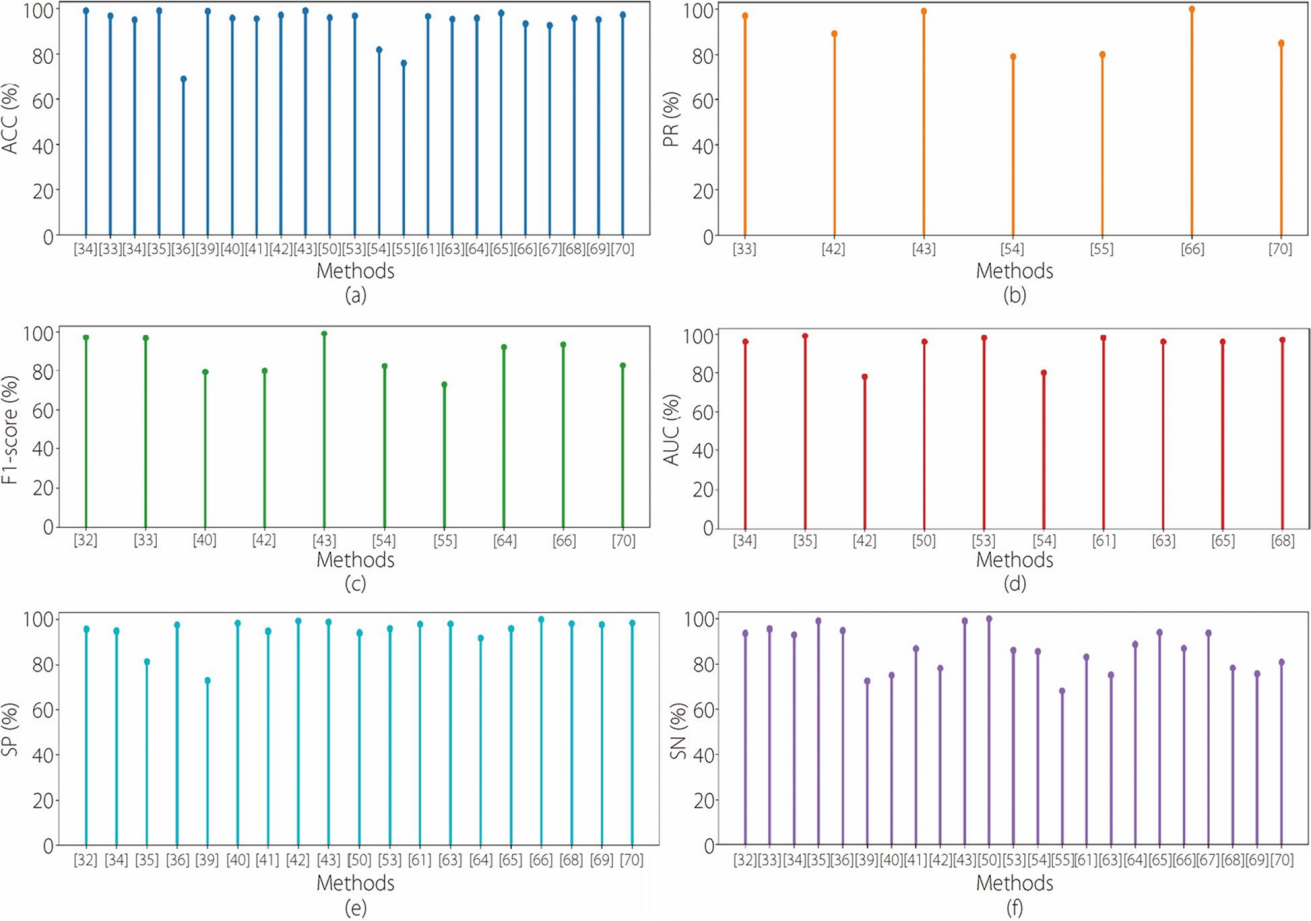
Performance analysis metrics. **a** ACC; **b** PR; **c** F1-score; **d** AUC; **e** SP; **f** SN

Figure 7 shows the metrics used in the performance analyses of the various existing HR techniques. Overall, this review emphasizes the role of AI in enhancing the PR and effectiveness of HR diagnosis using advanced image analysis techniques. These developments not only improve the diagnostic capabilities, but also hold promise for enhancing patient outcomes by enabling earlier detection and tailored treatment strategies.

Table 7 presents a comparative analysis of various models to address the tradeoffs between model complexity and performance. Each model demonstrated a balance of computational time, misclassification rate, and memory usage, highlighting the practical considerations for clinical deployment.

**Table 7:** Assessment of different approaches

Method	Computational time (s)	Misclassification rate (%)	Memory (G)
CNN [7]	4747.11	6.5	-
Hyper mixed CNN [8]	4057.68	4.2	16
Explainable detection network [10]	1464	2.0	12
CNN [18]	1500	8.2	16
Fuzzy clustering [32]	13.61	3.85	16
EFCM [43]	10.23	2.0	16
HDR-EfficientNet [65]	184.5	4.0	16

In clinical applications, models must balance complexity with practical performance metrics, e.g., computational time and memory efficiency. For instance, although traditional CNN models (e.g., refs. [7, 18]) have long computational-times, methods, e.g., explainable detection networks [10] and EFCM [43] achieve significantly shorter inference times and higher accuracies, making them viable for real-time applications. Moreover, models with reduced memory requirements, e.g., the explainable detection network [10], are particularly suitable for environments with limited computational resources.

Models with lower computational demands and memory usage combined with higher ACC are better suited for clinical systems that require timely and precise detection. Table 7 serves as a guide for selecting the models that best meet the clinical deployment requirements based on performance tradeoffs.

### Applications

This review aims to leverage AI in RIA for HR diagnosis. Advanced computational algorithms and ML techniques are explored to automate the detection and evaluation of HR signs in retinal images. Key applications include the following:

- Automated diagnosis: AI is used to detect specific signs of HR (e.g., arteriolar narrowing and hemorrhages) with high ACC and efficiency.
- Early detection and treatment: Early intervention is facilitated by identifying the subtle changes in the retinal vasculature that are critical for managing patients with hypertension.
- Severity grading: AI is used to grade the severity of HR stages from mild to malignant based on quantitative metrics, e.g., the AVR and vessel tortuosity. This approach supports healthcare providers in making informed decisions by providing objective metrics and classifications derived from AI-based analyses.

### Challenges

Although the proposed review suggests significant advances in HR diagnosis using AI, several challenges must be addressed.

Data quality and quantity: Ensuring access to large, diverse, and annotated datasets is crucial for training robust AI models. Variability in image quality and differences in image-acquisition techniques pose challenges to model generalizability.

Clinical validation: Validating the efficacy of AI models in real-world clinical settings is essential for gaining acceptance among healthcare professionals. However, bridging the gap between the AI research outcomes and practical clinical applications remains challenging.

### Handling variability in data quality across models

Table 8 provides a comparative analysis of the various preprocessing methods used across different models to manage the variability in data quality, including noise, incomplete data, and low-resolution images. These preprocessing techniques enhance the model performance by mitigating the common challenges in low-quality data. Noise reduction methods, e.g., Wiener filtering and morphological background subtraction, reduce random noise and preserve critical features in noisy images.

**Table 8:** Comparison of preprocessing methods for managing data quality variability

Reference	Work	Preprocessing method	ACC
[40]	RGB to gray conversion	Image converter	0.9534
[43]	Exclude the unimportant external elements, eliminate the issue of color distortion	Image cropping Color channel conversion	0.9900
[45]	Normalize image size and contrast	Normalization	0.9634
[54]	Noise reduction	Dealing with missing entries and unequal class distribution, encoding categorical variables	0.8200
[64]	Sharpen the image and isolate the features	Structural background elimination, Gaussian-based preprocessing filter, Heisenberg matrix	0.9582
[65]	Increase the size of datasets	Data augmentation	0.9812
[66]	Improve brightness	Histogram equalization	0.9333
[67]	Advance the contrast of the image	Contrast-limited adaptive histogram equalization	0.9255
[68]	Standardization Improve the SN	Gamma adjustment Normalization	0.9561
[69]	Noise reduction	Wiener filtering	0.9533

Image enhancement techniques, including histogram equalization and contrast-limited adaptive histogram equalization, increase the visibility of details at low resolution and optimize them for model training. Standardization and normalization techniques maintain consistency across image inputs, supporting the model performance despite variations in image quality. Data augmentation addresses data incompleteness by expanding the dataset and improving the model generalization.

In addition, handling missing values and imbalanced data increases robustness in scenarios with incomplete datasets. These preprocessing methods are effective approaches for enhancing the model robustness and ACC when encountering variability in data quality.

### Future work

Subsequent research in this area should be directed towards improving the robustness of AI algorithms for differential performance capabilities across various population groups and disease severities in patients. A multimodal combination of imaging data may improve AI models towards wider applications and comprehensive diagnoses at the individual level. The interpretability and transparency of a system are important for its actual adoption in clinical practice and scrutiny by regulators. The use of AI beyond diagnostics for predictive analytics and ad hoc treatments is promising. Partnerships among research, clinicians, and industry partners should be developed for the full transition of these technologies into practical clinical applications, optimal early interventions for enhanced patient outcomes, and a lower healthcare burden for HR. RIA for HR has already been revolutionized with the help of AI; however, ongoing innovation and collaboration across disciplines will allow this next evolution to be transformative within medicine.

## Conclusions

This review has discussed the revolutionary role of AI in the diagnosis and management of HR. It has also reviewed the state of the art in A/V recognition, AVR computation, vessel tortuosity quantification, and HR descriptor systems, as well as progress in retinal vessel segmentation algorithms and new biomarkers obtained from retinal images. In this respect, AI has demonstrated considerable potential through state-of-the-art RIA for the automatic screening and assessment of HR markers with high ACC, efficacy, efficiency, and robustness regarding classification error issues. By using advanced ML and DL, AI has the potential to improve the ACC of HR detection. Introducing AI into this area will likely lead to earlier detection, and thus, better HR management, which is a cornerstone for boosting clinical endpoints. Modern advances and evolving trends in RIA supported by AI heralds great promise and merits further innovation, improvements, and expansion. AI will likely play a central role in the future of ophthalmology, and hence, provide major advantages to both healthcare providers and patient health with its evolution. In summary, the introduction of AI into HR diagnosis can enable faster and more accurate detection and treatment, ultimately providing better healthcare delivery and protection of human life.

## Data Availability

Data sharing is not applicable to this article, as no datasets were generated or analyzed in the current study.

## Abbreviations

HR: Hypertensive retinopathy
AI: Artificial intelligence
A/V: Artery-vein
RIA: Retinal image analysis
DL: Deep learning
U-Net: U-network
ML: Machine learning
SVM: Support vector machine
AVR: Arterio-venous ratio
CNN: Convolutional neural network
IoT: Internet of things
AC: Accuracy
SN: Sensitivity
SP: Specificity
PR: Precision
AUC: Area under the curve
NN: Neural network
MFOA: Micro firefly optimization algorithm
EFCM: Enhanced fuzzy c-means
IDRiD: Indian diabetic retinopathy image dataset
DRIVE: Digital retinal images for vessel extraction
STARE: Structured analysis of the retina
HRF: High-resolution fundus
RNFL: Retinal nerve fiber layer
HER: Electronic health records
PLRS-Net: Pool less residual segmentation network
CHASE-DB1: Child heart health study in England data base1
TP: True positive
FP: False positive
FN: False negative
TN: True negative
RVM: Retinal vessel morphometry
